# The Indian Rebellion in Its Moral and Psychological Aspects

**Published:** 1858-01-01

**Authors:** 


### Art. II.—THE INDIAN REBELLION IN ITS MORAL AND PSYCHOLOGICAL ASPECTS.

The operations of the mind and the play of the passions on an extensive scale, are among the most interesting topics of Psychology. They are uncommon, because it is but seldom that the routine of life is diverted from its accustomed channels, or broken up into fragments so as to eliminate its component elements, and reveal the essential spirit by which it is animated and impelled. For the most part, society proceeds in a placid, if not an artificial way; its temper, however restless or energetic, is subdued by a code of conventional laws; and the natural impetus by which it would be incited to action, were it to yield to its own instigations or caprice, is held in check by the pressure of surrounding circumstances, or the force of public opinion. Thus, the wayward youth is overruled by domestic discipline, and the populace of a large community is overawed by a rigid system of police. But on occasions of public or private disaster, the inward man breaks out and unconsciously betrays himself. Those propensities and that disposition which would, under ordinary affairs, have remained latent and unperceived, suddenly come into view and startle us by the novelty of their vice, on the one hand, or their virtue, on the other. It would seem that we do not know with whom we are associated, nor what we ourselves really are, till some change of fortune draws aside the veil and discloses the inmost workings of the soul, both in our own breasts and those of others. The psychologist has frequent opportunities of verifying the justice of this remark. Of the overthrow of the intellect, which it is his peculiar office to manage and relieve, individual cases of this description are continually presenting themselves to his notice. But they are individual, not social cases; private, rather than public; local, and not universal. The opportunities of observing the last kind, as in great political revolutions, or the disastrous retreat of vast armies before an overpowering foe, or during violent monetary panics, when attacks of insanity are no unusual consequences of horrors witnessed or losses sustained, are, happily, few and far between in the course of our lives. And yet, though their moral effects are too well known for more than a passing allusion to them, they do not comprise the whole of the question. There are, besides, the distinctive differences of personal character, of moral worth or obliquity, of intellectual strength and weakness, and of peculiarities, finally, of race, religion, education, and innate propensities. These discordant elements can be separately examined only on the occurrence of signal catastrophes; and their examination must needs be hasty and brief, because their manifestation is nothing more than the transient ebullition of the moment. But transient and evanescent though they be, they are, nevertheless, both the absolute and substantial endowments of mankind, proper to the hour of peril and the day of calamity. They constitute the heroic, in the highest sense of the term; they test the metal we are made of; they cost the life and name of the actors; they ennoble or vilify a family, a class, or a clan; they make or unmake a nation! This is the study of psychology on the world's stage—the analysis of the human understanding in the living drama of races and creeds. Such is the theme that has engaged our attention while contemplating the Indian mutinies, of which we now proceed to present our readers with a narrative, comprising a comparative review of its salient incidents, historical bearings, and philosophic relations.

The European inhabitants had slumbered peaceably on the night of the 10th, and the large cantonments, garrisoned exclusively by native troops, were undisturbed on the morning of the 11th of May, when some cavalry troopers dashed across the bridge of boats on the Jumna, and entered the city of Delhi. They left half their number to hold the bridge, while the rest—not more than fifteen, it is said—galloping through the streets, created a riot as they passed along among the scum of the population,—faced the 54th Native Infantry, which was ordered down to resist them,—pistolled the Colonel and European officers, who were without their arms,—and in an incredibly short space of time took military possession of a fortress and arsenal the strongest and the best appointed in India, and obstinately continued to hold it till the 20th of September following. All the Christian population within its walls were massacred. A very few only escaped to tell the tale. The chief military officers fell; the banks, printing houses, and other non-military stations, were plundered, the inmates murdered; and a vast number of clerks, half-castes, inferior Europeans, and every real or nominal Christian, slaughtered on the spot. No massacre took place in the cantonments; in fact, the regiments left there seem to have stayed with their officers the greater part of the day, and only gradually or passively went over or refused to act. The officers and ladies were collected at one point, and long and anxiously did they expect the arrival of aid from Meerut. Mutineers in abundance arrived thence; but the day wore away, and no Europeans came. Some thoughtful mutineer—whether in mercy or in triumph, we know not—sent up a cart and deposited before the survivors the bodies of the officers killed in the city. Bitter, indeed, must have been the feelings of those who remained. In the evening, finding themselves deserted by their men, and without aid from Meerut, they determined to fly.

They had a number of carriages of different kinds, and they generally escaped in safety. But other stragglers from various other quarters of the place had more difficult and perilous escapes, wandering as fugitives from village to village; and some of them were thus wandering for several weeks before they reached their European relatives, and enduring all the time most cruel hardships of want and weather.*

Thus ended the day on which Delhi fell, and with its fall quickly followed that of every other military station in Upper Bengal—Benares, Allahabad, Agra, Lucknow in Oude, Cawnpore, and others. Regiment after regiment revolted, and, with different episodes of bloodshed and cruelty, passed over to the side of the rebels, and turned their arms against their employers, and the white population generally. The first shot was fired on the 10th of May, at Meerut, and by the 10th of June the whole of the North-west Provinces had become completely revolutionized; the British rule was confined to a very few insulated stations held by European troops; in the country generally it had ceased to exist. Entire anarchy had taken its place.† Dinapore and Arrah revolted at a later date. Some local insurrections broke out in the Bombay Presidency; and the Punjaub was prevented from following the same desperate course by nothing else than the decision of its energetic Commissioner and the presence of several European regiments. In all other quarters, the rebellion was complete; and by the beginning of July we had 100,000 well-disciplined troops of all arms—cavalry, infantry, and artillery—arrayed against us in the field, or in actual possession of the strongholds of Delhi and Cawnpore, if not of Lucknow, Agra, Benares, and Allahabad. Never was any military achievement so well planned, or so well achieved without a plan; never was a civilized Government so easily taken by surprise, or, if not surprised, so easily wrested of its strongest stations and richest provinces, against its will; its officials slain, its people tortured and killed, its highest and lowest military officers put to death or to flight, its houses and cities burnt to the ground, and its treasure plundered and carried off in triumph or contempt. The rebellion was a master-stroke; it had attained its first object by a dash, and in the short space of a month it had inflicted a wound on the name and character of the British power which it will take years to heal up, more than half a century to re-establish in its pristine strength, and, it may be, never to recover from entirely. At all events, it can never be forgotten by either side; suspicion must always re-

* The *Times,* October 24th, 1857. History of the Bengal Mutineers, from Umballah.

† The *Times,* October 26th.

main, and with suspicion come also distrust, retaliation, and revenge. As we stand by and look on, we are tempted to exclaim, Alas! that in one hour so great riches should have come to nought!

In the foregoing brief account, our minds are carried back to the ruthless period of Tamerlane, who once proposed to exterminate all the inhabitants of the Chinese Empire, and turn it into a sheep-walk. From this direful project he was hindered by death; but a similar process was his rule with the cities he conquered.* Let it be understood, he came down upon cities living in peace and prosperity, like those in India,† which had done him no harm, which had not resisted him, which submitted to him at discretion. He sacked the city, killed or enlisted the soldiery, and divided the rich, the women, and the artisans, among his followers. The Tartar character has been always the same, and in the Indian mutinies we see it exemplified to the very letter.

Our object in selecting this popular theme for our Journal, is on account of the very striking manner in which certain emotions of the mind have displayed themselves on so many trying occasions—chivalry, devotion, and fortitude, on the one hand; cowardice, treachery, and cruelty, on the other. And these noble or ignoble qualities have not been the transient outbursts of the moment, but, on the contrary, the inborn energies of the soul, which belong not to the individual, but to his race, and not merely to his race, but to the political, moral, and social institutions of which he is the living representative and agent. We are accustomed to delight ourselves with tales of romance, and the spirit-stirring scenes of the great drama of Shakspeare or Racine; but within the last few months fiction has been surpassed by truth, and tragedy fades into insignificance before the actual horrors endured by our countrymen, or resisted to the last by their own heroïc bravery, or coolly narrated by their masterly pens in the midst of danger. Nor have our countrywomen fallen short of their high birth and destiny. Their courage has risen with the occasion, and shone the brighter, the darker grew the storm. They never quailed. They literally stood to their guns. They faced the shower of bullets—the glare of the naked tulwar. They loaded the muskets or pistols, and handed them to their husbands for their mutual defence, in the midst of the hottest fire; nay, more, when all was done that could be done

* Neither age, nor the severity of winter, could retard the impatience of Timour; he, mounted on horseback, passed the Sahun (Iaxartes) on the ice, marched 300 miles from his capital, and pitched his last camp at Otrar, where he was expected by the angel of death. Fatigue and the indiscreet use of iced water accelerated the progress of his fever, and the conqueror of Asia expired in the seventieth year of his age: his designs were lost, his armies were disbanded; China was saved.—*Gibbon.*

† Newman's "Turks," p. 40.

by human strength and self-possession in the front of overwhelming forces, they gave up their lives rather than their honour, and devoted themselves to death instead of infamy. We see a noble Englishman and his wife defending a tower. The woman loads her husband's pistols: he fights the ruffianly legion till it swarms up the walls. Then the brave fellow, kissing his wife, slays her—which he does for her sake, but which a Rajpoot would have done for his own—and then he kills himself, and they die as the infernal pirates rush upon them. Instead of the anecdote of Chelonis and Clœlia, our children may in future learn, from the victims of Bengal, how nobly an Englishwoman may die—how Mrs. Skene fought by her husband's side, how Miss Jennings offered to sacrifice herself to save her father. And upon these countrywomen of ours—matrons, virgins, tender girls—and upon tender children, have been practised the vilest tortures; things to be remembered, not descanted upon; infamies which the Greeks would have called The Unutterable, failing to describe them.* Nor are the instances of high devotion only a few. At Futtyghur, the wife and child of Mr. Tucker being about to fall into the hands of another rebellious set, she called to her husband to shoot her at once. He did so, his child also, and then himself. A Major Robertson also shot his wife and children and himself, under similar circumstances.† This is a new and melancholy feature in the history of these tragedies.‡ But everything of the sort falls into the shade compared with the lofty and moral grandeur of the heroine of Cawnpore.§ A daughter of General Sir Hugh Wheeler's was taken off by a sowar, and put into his house along with his wife, near the church. This girl remained till nightfall, and when he came home drunk and fell asleep, she took a sword and cut off his head, his mother's head, the two children's heads, and his wife's, and then walked out into the night air; and when she saw other sowars she said, "Go inside and see how nicely I have rubbed the Resseldar's feet!" They went inside, and found them all dead. She then jumped into a well, and was killed. There is a stern brevity in the tale that strikes us with mixed admiration and awe; and in those few calm, biting words, "I have rubbed the Resseldar's feet," there is a horrible irony, expressive of the

* The *Leader*.

† Let us hear both sides of the question:—"I have given up walking about the back streets of Delhi, as yesterday an officer and myself had taken a party of twenty men out patrolling, and we found fourteen women with their throats cut from ear to ear by their own husbands, and laid out in their shawls. We caught a man there who said he saw them killed, and showed us their husbands, who had done the best thing they could afterwards, and killed themselves."—*Extract of a letter from Delhi after the storming of it*:—The *Times*, November 19th, 1857. So that what is heroic on our side is dastardly on theirs.

‡ Letter from Jubbulpore, August 5th. § The *Times*, October 16th, 1857.

frenzy of passion at its highest pitch of exaltation. Those who listened to these words, and saw what this girl had done, were seized with fear, and none of the rebels would have anything to say to the English women, whom the Nana at first proposed to give over to the soldiery. It was Medea again, but without her magic; Judith and Holofernes, in the midst of drunken carnage; Virginius and his daughter, in the Roman Forum; Tarquin and Lucretia. But no, it was none of these, for they all fail in the comparison, and that tender girl stands alone in her self-devotion—THE BRITISH SOLDIER'S HIGH-MINDED DAUGHTER. Alas! alas!—so young, so fair, so pure—to have died so early—to have perished thus! In the history of India, her name will descend to the latest posterity. A hundred and fifteen women and children were imprisoned with scarcely any food for six days, except gram and such stuff. The Nana ordered them to be killed. When they learnt this, the ladies tore their clothes, and with the shreds fastened the door. A sowar jumped over the wall, and began the slaughter; other sowars came through the doors, and all the prisoners were killed. Their bodies were cast into the well. Twenty-five women and children remained alive under the heap of dead bodies—the women were killed, and the children dashed against the ground. The floor of the apartment was clotted with gore. Portions of dresses, collars, and children's socks, and ladies' round hats, lay about saturated with blood; and in the sword cuts on the wooden pillars of the room, long and dark hair was carried by the edges of the weapons, and there hung their tresses. "I often wish," says a writer who visited this room upon the recapture of Cawnpore by General Havelock, shortly afterwards—"I often wish I had never been there. Their limbs may be seen sticking out of the mass of gory confusion down the well into which they were thrown. I picked up a mutilated prayer-book. It had lost the cover, but on the fly-leaf is written, 'For dearest mamma, from her affectionate Tom, June, 1845.' The Litany, at page 38, is sprinkled with blood!" Some portions of a diary written in pencil were found, and it broke off at the day of the massacre, or just before; and a small work-box was open, and the things scattered about in the midst of the blood. General Neill compelled the high-caste Sepoys, whom he caught, to wash up the blood, and afterwards hung them, by both of which acts they defiled themselves and lost their caste; and the 78th Highlanders, on their arrival at Cawnpore, extricated General Wheeler's daughter from among the mangled remains, gently removed the ringlets from her head, separated a portion of them for her friends at home, and divided the remainder equally among themselves, each man swearing, as he received his share, that for every hair he had received a Hindoo should die by his hand. Well might Lord Palmerston exclaim, on the 9th of November, at Guildhall, "Henceforth the bravest soldier may think it no disparagement to be told that his courage and his power of endurance are equal to those of an Englishwoman!"

The atrocities of the Nana Sahib at Cawnpore are, however, only in keeping with his creed and race. At the capture of Sivas by Timour, in 1389, four thousand Christian warriors were buried alive by his orders. Their heads were tied down by cords lashed tightly round the neck and under the thighs, so as to bring the face out between the legs. In this agonizing posture they were thrown into graves, purposely left open for a time to prolong their misery.* After the battle of Nicopolis, Bajazet sat on a lofty throne to see the execution of three hundred Christian gentlemen, pitilessly murdered before his eyes. The meaner captives were sold for slaves, and the women, who had followed the French camp, were abandoned to the brutality of the Turkish soldiers.† The Sultan Musa, in 1424, caused the carcases of three Servian garrisons to be arranged as tables, and a feast spread upon them, at which he entertained the generals and chief captains of the Ottoman army; and in the memory of many of us, on the insurrection of the Greeks in Scio, their barbarian masters carried fire and sword throughout that flourishing island till it was left a desert, hurrying away women and boys to an infamous captivity, and murdering youths and grown men, till out of 120,000 souls in the spring time, not 900 were left there when the crops were ripe for the sickle.‡ In the present year the same scenes have been repeated in India, and the daily journals are teeming with their horrid particulars. We see a young girl, naked, mounted upon a cart, paraded through the streets of a city, subjected to the last humiliations, and torn limb from limb by gangs of black satyrs.§ Give full stretch to your imagination, says a writer who had witnessed what he describes, —think of everything that is cruel, inhuman, infernal, and you cannot then conceive anything so diabolical as what these demons in human form have perpetrated. A man who witnessed the last massacre at Delhi gives a horrid account of it, stating that little children were thrown up in the air, and caught on the points of bayonets, or cut at as they fell with tulwars. One lady was stripped, abused, and murdered in the most cruel manner, first cutting off her breasts. Another lady, who had hid herself under a bridge, was treated in the same manner, then hacked to pieces, and her mangled remains thrown out on the plain. We found

* Creasy's "Ottoman Turks," vol. i. p. 74.
† Ibid., pp. 60—84; on the authority of Froissart.
‡ Newman's "Turks," p. 136. § The *Leader*.

a pair of boots, evidently those of a little child, with the feet in them. They had been cut off just above the ankles. Children were put to death and cut to pieces before their parents, who were lashed to a post or tree ; and pregnant women were violated and ripped open before their husbands' faces, or flayed alive, or roasted over a fire, or slowly lacerated till they died. There is no end of their horrible ingenuity in torturing their victims. Bungalows were burnt to the ground—costly furniture broken and despoiled—silver services, money, and jewels stolen or strewed about. There was nothing but fire and blood, ruin, and desolation, and woe.

We might suppose that a new era had commenced in the history of the world, so regular and peaceful has been the course of the last quarter of a century. But peace and prosperity are not the rule of life. The historian knows but too well that his page recounts a wearisome catalogue of contest and misery from the first centuries to the last. Scarcely a hundred years have elapsed since the first French Revolution, whose enormities staggered the world; not fifty since the spoliation of Poland by Russia. At the close of the last century, to select one instance out of many, when the Russians took Oczakoff, in 1788, the same scenes were enacted. The Turks of Oczakoff had, before the siege, surprised a Russian village in the vicinity, and mercilessly slaughtered all the inhabitants. Potemkin and Suwarrow* caused the Russian regiments that were to assault the town to be first led through the village as it lay in ashes, and with its streets still red with the blood of their fellow-countrymen. With their natural, stubborn, savage courage, thus inflamed by the longing for revenge, the Russians advanced over the frozen Liman against the least fortified side of the city. Whole ranks were swept away by the fire of the besieged ; but the supporting columns still came forward unflinchingly, through musketry and grape ; four thousand Russians fell, but the survivors bore down all resistance, and forced their way into the city, where for three days they revelled in murder and pillage. No mercy was shown to age or sex ; and out of a population and garrison of forty thousand human beings, only a few hundreds (chiefly women and children) escaped, whom the exertions of the officers in the Russian service rescued from the indiscriminate fury of the soldiery.

Mr. Eton, who was with Prince Potemkin at Oczakoff, describes a touching scene which he witnessed there, and which he

* Suwarrow was the general to whom Prince Potemkin gave the laconic order,—"You will take Ismail." Suwarrow took it, but at a cost of carnage and crime unparalleled in the records of ancient or modern warfare. After his victories—for he was never defeated—Suwarrow used to retire to his tent and cry like a child.

cites as a proof of the fortitude and resignation, bordering on apathy, with which the Turks bear evils of the greatest magnitude. He says:—"The Turkish women and children (in number about four hundred) who were brought out of Oczakoff, when the city was taken, to the head-quarters of the Russian army, were put all together the first night under a tent, as no better accommodation could, under the pressure of circumstances, be made for them, though it froze exceedingly hard, and they suffered dreadfully from cold and nakedness, and many from wounds. As I spoke Turkish, I had the guard of that part, and the superintendence of them for that night. I observed that there reigned a perfect silence among them; not one woman weeping or lamenting, at least loudly, though every one, perhaps, had lost a parent, a child, or a husband. They spoke with a calm and firm voice, and answered the questions I put to them apparently without agitation. I was astonished, and knew not whether to impute it to insensibility, or the habit of seeing and hearing of great vicissitudes of fortune, or to a patience and resignation inculcated by their religion; and to this day I am equally unable to account for it. One woman sat in a silent and remarkably melancholy posture, insomuch that I was induced to offer her some consolation. I asked her why she did not take courage, and bear misforture like a Mussulman, as her companions did. She answered in these striking words:—'*I have seen my father, my husband, and my children killed; I have only one child left!*' 'Where is it?' I asked her with some precipitation. '*Here!*' she calmly said, and pointed to a child by her side, which had just expired. I and those with me burst into tears, but she did not weep at all. I took with me that night into my warm subterranean room as many of those miserable women and children, wounded and perishing with cold, as it would contain; they stayed with me twelve days, during all which time none of them either complained aloud, or showed any signs of excessive internal grief, but each told me her story (both young and old) as of an indifferent person, without exclamation, without sighs, without tears."*

The Russians are Mongol Tartars, and the Turks are originally the Tartar descendants of the White Huns of Sogdiana, or Bokhara; and cruelty is proper to them both. But in the sad tale just told by Mr. Eton, we see the kind-hearted Saxon, full of warmth and compassion; while the sublime apathy of the Turkish women stands out in fine contrast to the animation, courage, and tenderness of the English women under similar circumstances.

The Anglo-Saxon spirit again comes into view in the following incident, related, in a letter from India, by a soldier of the 78th

* Creasy's "Ottoman Turks," vol. ii. p. 291.

Highlanders to a relative in Perth:—"We came to a village and set it on fire—I saw nothing but flames around me. I came across a woman about twenty-two years of age, sitting over a man that, to all appearances, would not see the day out. She was wetting his lips with sistè. The fire was coming fast, and the houses all around were in flames. Not far from this I saw four women. I ran up to them and asked them to come and help the sick man and woman out, but they thought they had enough to do, and so they had, poor things; but to save the woman and dying man, I drew my bayonet, and told them if they did not, I would kill them. They came, carried them out, and laid them under a tree. I left them. The flames were in the clouds. I went to the other side of the village, and there were about one hundred and forty women and about sixty children all crying and lamenting at what had been done. The old woman of that small family that I had previously taken out, came, and I thought she would have kissed the ground I stood upon. I offered them some biscuits from my rations," &c. &c.*

In reflecting on the causes of so great a calamity as that of the loss of one-third of an empire in so short a space of time, we are naturally led to inquire what was the state of the public mind, as well as that of the Government, both political and military, at the moment of the outbreak. Could it have been prevented? Was it unforeseen? Did it happen without warning? The matter seems to have had the anxious care of the Government; but the Commander-in-Chief took it more easily, and was out shooting, far from the telegraph. He was at Umballah in March, and thence went to the hills, to Simla. It was known that there had been a disturbance at Barrackpore and Berhampore, and afterwards at Umballah; and night after night isolated fires occurred, as early as April. All were equally puzzled and confounded by what they saw and heard. People gossiped or laughed; and the Commander-in-Chief was distracted by the most opposite opinions. In short, nothing was done; and Delhi was in the possession of the mutineers before any one was aware of it. There is no doubt that if a European force, however weak, had been marched into Delhi at the first intimation of alarm, that that fortress at least might have been preserved. The officers in command at Umballah were Queen's officers; there were also some dragoons and a battalion of infantry at Meerut. Had only the wing of a regiment been opposite the magazine—had only a few dragoons been on guard at the bridge of boats on the fatal morning of the 11th of May, the revolt might have been averted, if not suppressed. But the chief military oversight was at Meerut. At that station

* *The Times*, October 24, 1857—"History of the Indian Mutinies." This clever *resumé* occupies several columns in two impressions of the paper.

the European forces were stronger than at any in India; but the regiments of European cavalry and rifles and large artillery were rendered inefficient by the general in command. Even in cantonments there was no effective opposition made to the first beginnings of the revolt; the mutineers were allowed to depart unmolested; and the active pursuit that might and ought to have been followed up to the Water-gate at Delhi, was omitted; the lucky moment was lost, and with it was also lost the whole of the north-west provinces of Bengal, Lucknow, and the newly-annexed kingdom of Oude.*

A panic, likewise, was prevailing at the time, and it certainly tended to render all arrangements more difficult. You could believe no one. People seemed to have lost the use of their senses; and those whom you would have implicitly believed at any other time, now told the most wonderfully unfounded stories. In short, there was no end of *canards*. It was impossible to distinguish truth from falsehood—a palpable sign of moral and intellectual debility commonly observed on most occasions of alarm and danger, whether social or individual. And there was no superior mind, cool and collected, in a position of authority equal to the emergency. The same want of foresight and precaution would have lost London on the 10th of April, 1848, as it had already lost the throne of Louis Philippe at Paris in the preceding February.†

One, and only one, solitary instance of determination and tact occurred at Delhi at that critical moment.‡ The Subahdar of the guard on duty at the magazine informed Lieutenant Willoughby and me that the King of Delhi had sent down word to the mutineers that he would, without delay, send scaling-ladders from the palace for the purpose of scaling the walls, and which shortly after arrived. On the ladders being placed against the wall, the whole of our native infantry deserted by climbing up the sloped sheds on the inside of the magazine, and descending the ladders on the outside, after which the enemy appeared in great numbers on the top of the walls, and on whom we kept up an incessant fire of grape, every round of which told well, as long as a single round remained. Previous

* *The Times*, October 24th, 1857, p. 8, sixth column.

† There are two sides to every question. "Let us for a moment suppose that the mutiny had at its first outbreak been quelled, subsequent events and disclosures have only proved to us that the outbreak was premature, and that had it been overcome at the time, it would probably have come upon us at the appointed day with a force that none of us could have hoped to withstand."—*Letter from an Engineer Officer, September* 28: *The Times*, November 24, 1857. Because the outbreak was premature, it was no reason for not resisting it at the first. The first blow might have been decisive. *Obsta in principiis*, is a universal axiom.

‡ Official account, by Lieutenant G. Forrest, of the Ordnance department.

to the natives deserting us, they hid the priming pouches; and one man in particular, Kurreemburkh, a durwam, appeared to keep up a constant communication with the enemy on the outside, and keep them informed of our situation. Lieutenant Willoughby was so annoyed at this man's conduct that he gave me an order to shoot him should he again approach the gate.

Lieutenant Raynor, with the other Europeans, did everything that possibly could be done for the defence of the magazine. Conductor Buckley, assisted only by myself, loaded and fired the several guns in rapid succession, firing at least four rounds from each gun, and with the same steadiness as if standing on parade, although the enemy were then some hundreds in number, and kept up a continual fire of musketry on us within forty or fifty yards. After firing the last round, Conductor Buckley received a musket-ball in his arm, above the elbow. I at the same time was struck in the left hand by two musket-balls, which disabled me. Lieutenant Willoughby gave the order for firing the magazine, which was at once responded to by Conductor Scully firing the several trains. The convulsion took place; and such as escaped from beneath the ruins—and none escaped unhurt—retreated through the sally-port on the river face. Lieutenant Willoughby and I succeeded in reaching the Cashmere-gate. What became of the other parties it is impossible for me to say.*

This spirited scene has had its counterpart on many other previous occasions in the art of war. Presence of mind in the midst of danger is the chief attribute of an officer in command. It is that which places him above the crowd, and makes him a distinguished man. It sheds a lustre round his name, and emblazons his deeds in the annals of fame. Three hundred years ago, a solitary tower resisted the Turkish batteries, after all the exterior defences had been destroyed.† The name of the place was Szigrette; the date, 1520; the commandant's name, Zriny; his force reduced to not more than six hundred men. The Janissaries advanced to the assault along the bridge

* Died, on the 10th of May last, of his wounds, in the 28th year of his age, Lieutenant George Dobson Willoughby, Bengal Artillery, and Commissary of Ordnance at Delhi, eldest son of the late George Thomas Railton Willoughby, Esq., of Bath, and grandson of the late Dobson Willoughby, Esq., of Belsize, Hampstead. It is supposed (for there is no authentic account) that after his gallant devotion in the defence and destruction of the magazine at Delhi (on the morning of the 10th of May last), although so desperately wounded and scorched as to be hardly recognised, he endeavoured to make his escape to Meerut (with three or four other officers, who had joined him on his way), but, exhausted by his wounds, he fell an easy victim to some miscreants of a village, who murdered him in his resisting their attempts to plunder him, and whilst defending one of his companions.—*The Times*, November 25th, 1857.

† Creasy's "Ottoman Turks," vol. i., p. 312.

that led to the last shelter of the defenders. Zriny felt that his hour was come. He attired himself as if for a festival; a diamond clasped his plume to his helmet; his falchion, with the keys of the fortress and a purse full of choice ducats, hung from his glittering baldrick. "He who lays me out," he exclaimed, "shall be paid for his trouble; the keys I surrender only with my life!" The banner of the Empire was borne before him. He descended to the outer court. The Turks were at the gate. A mortar, heavily loaded with missiles, was brought to bear upon the entrance. As the foremost Janissary raised his axe to break in the door, the gates were flung open, and Zriny himself fired the mortar. The deadly shower poured through the assailants; and amidst the smoke, din, and confusion of this unexpected carnage, Zriny sprang forward sword in hand, followed by his devoted few. Six hundred Magyar sabres drank deeply of Turkish blood on that fatal day. Zriny, fighting to the last, fell pierced with two musket-balls through his body, and an arrow-wound in his head. The Ottomans, with a shout of "Allah" at his fall, pressed into the citadel, fired the place, and began the plunder. The fire caught a train, purposely laid by Zriny from the entrance to the magazine, which instantly exploded, and destroyed three thousand of the destroyers along with the battlements and walls of the fortress. Solyman the Magnificent, who had set himself down before Szigrette in full expectation of success, had died during the siege. He lay stark and cold in his tent, while the trumpets flourished aloud the note of victory, and the flag of earthly pageantry waved in vain above his senseless corpse. The world and its glories had closed above the career of one who had shaken the powers of Christendom to their centre, and who had owned the obedience of all the most celebrated cities of biblical and classical history, except Rome, Syracuse, and Persepolis. The Tigris, the Tanais, the Borysthenes, the Danube, the Hebrus, and the Ilyssus, had rolled their waters within the shadow of the Horse-tails. But a change had passed across the spirit of the dream; the pomp was over, and both Zriny and Solyman were numbered with the dead.*

The morning broke clear and bright over the plains of Hindostan, the day after the first thrilling atrocities at Delhi, and

* The fall of Szigrette suggested to the mighty Byron the idea of his highly animated poem, "The Siege of Corinth:"—

"'Tis fir'd!
Spire, vaults, the shrine, the spoil, the slain,
The turban'd victors, and the Christian band;
All that of living or dead remain,
Hurl'd on high with her shiver'd fane,
In one wild roar expir'd!"

the city of the Moguls outwardly appeared as serene as it had done the day before. Nothing betokened the change that had occurred within its walls; and none, save a few wandering and dispirited outcasts, guessed the heavy blow that had been struck at our Anglo-Indian empire, the name and *prestige* of England. "We started from Delhi," says one writer, "with five ladies and four officers besides myself; but afterwards in our wanderings fell in with two sergeants' wives and two little children, with two more officers and a merchant; so altogether, on coming into Meerut, we were about seventeen souls. Oh, great Heaven! to think of the privations we endured, and the narrow escapes we had! We used to ford streams at night, and then walk on slowly in our dripping clothes, lying down to rest every half-hour; for some of the ladies were wounded, and all so fatigued and worn out they could scarcely move. Had we been by ourselves, we should have made a dash for Meerut at once; but having these unfortunate women with us, what could we do? Sometimes we heard the villagers combining to murder us, and the whole time were in dread of being pursued and killed by the mutineers from Delhi. At one time we were attacked by the villagers, and robbed of everything we possessed: had we not had the ladies with us, we would have fought for it, and sold our lives dearly, instead of quietly giving up our arms as we did; for we had a few blunt swords among us, with one double-barrelled gun." A lady, writing of her husband, says—"He found it was all over with him, and so he rode off, and got into a jungle, where he stayed a great part of the day. A groom, who had got his mare at a village, tried to betray him; so he rode off again. Only fancy how dreadful it was for him, wandering about in the heat of the day, not knowing where to go, and begging for a little water, or drinking it out of the little streams, he was so thirsty." "As we were getting into the boats," writes a third, "we saw the Sepoys plundering the Treasury. On reaching a place about three miles below Fyzabad, we met some mutineers. There were 800 or 900 of them. About 100 of them fired on us when we were 100 yards off. We pushed off to the opposite bank of the Ganges, and got on an island among some jhow fields. The mutineers got into dingies and followed us. There were about thirty or forty yards of water between us. The major was drowned." A fourth writer states:—"There was a small deep river behind the house, and beyond the river a jungle of cypress and brushwood. Some escaped by the ford. I came up with a lady struggling to get on with her little child in her arms—a girl two and a half years old—and her husband with her, carrying a boy about six months old. I took the child from her arms, and with the aid of a quarter-master-sergeant, got it safe and sound, all three escaping unscathed through the fearful shower of bullets sent after us as we crossed the river and hid ourselves in the friendly jungle. The father and mother, with the baby, were killed."

"The Nana Sahib declared, 'We never deceive; if we do, God will judge.' The General said, 'If you intend to deceive me, kill me at once; I have no arms.' The Nana replied, 'I will not deceive you; rely on us. I will supply you with food, &c., and convey you to Allahabad.' On this the General went inside the entrenchment, and consulted with the soldiers. They said, 'There's no reliance to be placed on natives—they will deceive you;' a few said, 'Trust them; it is better to do so.' On this the General returned, and said, 'I agree to your terms. See us away as far as Futtehpore; thence we can get easily to Allahabad.' The reply was, 'No, sir; I will see you all safe to Allahabad.' On this, twenty boats were ordered with covers to them. When the Nana saw all was settled, he said, 'Don't let the treasure be taken; send that to me.' The General said, 'You may have the money;' there were three lacs in cash at this time. The Nana said, 'You breakfast on board the boats at ten A.M. to-morrow, and dine on board, and leave the entrenchment clear by eleven A.M.' The General assented to this. They were all ready, when a message from the Nana came, saying, 'The boats will not be ready to-day; you must leave to-morrow; leave in the evening.' The General said, 'I wont leave at night, as you may play us false.' The Nana said, 'Very well; leave at four P.M.' On the following day, the Nana took away all the treasure. At this time the delay again took place in their departure; all the ladies and children were dressed and ready. The General asked the Nana, 'Are all our servants to go with us, or do you supply us with servants?' The reply was, 'Yes.' On the following day, though suspicions were entertained of the faith of the Nana's party, still they hoped all was right. The Nana sent on Sunday to say the servants were not to go, as the ladies and women could look after themselves. On this being heard, they were all alarmed. At seven A.M. the mutineers surrounded the entrenchment, and all the Englishmen in their power; the servants ran away and were cut down; a few escaped; all were alarmed. The rebels reached (?) the entrenchments, and said, 'Come to the boats; all is ready.' Ladies and children were sent on elephants, dhoolies, &c., and the men marched to the river, and then embarked on the boats. When they all saw food prepared and all comfortable, they were delighted. When a few had gone on board, and others were waiting to embark on the river-side, a gun opened on them with canister (this gun and others had been masked); one boat took fire, and then another gun opened, and four boats were fired; on this, those who escaped the fire jumped into the water. The Sepoys also fired muskets; the Sowars entered the water on horseback, and cut numbers down. Fifteen boatloads of English were massacred; one hundred and eight women and children escaped this massacre, but many of them were wounded. The Nana said, 'Don't kill these; put them in prison.' The boat containing General Wheeler and other ladies and gentlemen got off for twenty-two miles, when they were seized by the Zemindars of Joagnuhar, and had their hands tied behind them, and were taken back to the Nana. Mrs. Read, Thomas Greenway, Mrs. Kirkpatrick, Mrs. M'Kenzie, and Captain M'Kenzie, and Dr. Harris, and several Europeans, were among the party. The Nana was much pleased. Owing to the General's old age, he said, 'Loosen his arms.' Hoolar Sing, kotwal of Cawnpore, said, 'Don't do so.' The Nana said, 'Take them to the guard, and let the others remain where they are.' One Sepoy and Sowar killed each an European. Dr. Harris was wounded with two balls, and then addressed the rebels—'Shoot me, or kill me; my countrymen will revenge my death before long.' Two Sowars then cut him down, and he died."—*The Times*, October 16, 1857.

The treachery at Cawnpore is an instance of the Mohammedan character towards friend and foe. After Selim the Terrible had entered Cairo, in 1517, without opposition, and the garrison which he placed in it had been slain to a man by the indomitable Touman Bey, he endeavoured to retake it with some of his best troops. A street fight ensued, but the Mamelukes retained possession of the place. Selim then proclaimed an amnesty to such of the Mamelukes as would surrender. On the faith of this promise, eight hundred of the chief Mamelukes surrendered themselves, or were given up by the citizens. Selim had them all beheaded, and then ordered a general massacre of all the inhabitants of Cairo. 50,000 human beings are said to have perished in this atrocious butchery. One of the titles of the Sultans is that of the "Man-slayer," to this day. It is a canon law of their policy to destroy their nearest kin on mounting the throne—a prerogative they have exercised to the full without scruple or reserve. Amurath IV. is reckoned to have put to death 100,000 human beings in his short reign, from 1623 to 1640. He once ordered a party of dancing women to be seized and drowned; and at another time he ordered the batteries to open on a boat full of females passing along the Bosphorus, too near to the walls of the seraglio. It was his favourite maxim, that "vengeance grows grey, but not decrepit." Von Hammer gives a long list of the confidential officers put to death by Solyman the Great; and it is even stated by Turkish historians, that he inveigled his own son into the royal pavilion on purpose to have him bowstrung by surprise, and that, becoming impatient at the long-continued struggle between the Mutes and his victim, he himself also looked in on the horrible scene, and instigated them to the more speedy despatch of their work. A similar story is related of Selim I., who witnessed from an adjoining chamber the murder of his brothers, whom he had kidnapped for that purpose. The massacre of the Mamelukes at Cairo, in 1811, by Mehemet Ali, and that of the Janissaries at Constantinople, by Mahmoud II., in 1826, were both of them acts of the vilest treachery. So that the massacre at Cawnpore has nothing extraordinary in it, but is in strict keeping with the true spirit of the race.

Upwards of twelve centuries have elapsed since the contest between the Crescent and the Cross first began its mortal and obdurate strife. The years of the Hegira are fast running out; the last sands of its hour-glass are almost drained away; and yet the fatal four hundred years from the taking of Constantinople have already passed their close, and the Turkish and Christian prophecies respecting their awful accomplishment remain unfulfilled. The green flag of the Prophet waves above the Sublime Porte, and the illustrious line of the Osmanlis presides at Istamboul.* Severe as have been the Turkish reverses, both by land and sea, for the last two hundred and seventy years, and greatly as have been curtailed the frontiers of Turkey in Europe in the course of that long period, the Ottoman Princes nevertheless continue to hold the key of Europe in their hands, and to guard the passage of the Bosphorus with jealous pertinacity. The spirit of Islamism breathes in the desecrated sanctuary of St. Sophia, is proclaimed by the muezzin from the tops of their loftiest minarets and mosques, and is exhibited in the sanguinary and accursed tempers of the Mohammedan Sepoys or their miscreant chiefs, such as Nana Sahib. The hard struggle begun by Charles Martel, at Tours, in 732, and by Leo the Isaurian with his Greek fire, in 716, still goes on; and it has at length devolved on British valour to carry on the same deeds of arms in India at the present day, as those which once so effectually routed the Mohammedans before Vienna, in 1520; before the little fortress of Malta, so gallantly defended by La Vallete, in 1565; and before the fleets of Don John of Austria, at Lepanto, in 1571. In this light, the re-capture of Delhi, stained as it has

* "*Que diable faire de Constantinople?*" said Joseph of Austria to Catherine of Russia, joking over their plot for the destruction of the Ottoman Empire, while at Batchiserai, or at the Gulf of Atkiar, the modern Sebastopol, in 1787. "What shall we do with those poor devils the Turks?" The royal joke is as good as ever; for Constantinople is seventy years older than it was then, and, in spite of so many Russian treaties to the contrary, the Turks are at this moment the faithful allies of the Western Powers.

been with the blood of heroes, among whom is the distinguished Nicholson, who fell in the assault, assumes a character of renown far beyond that of the contest itself, and commensurate only with the records of the Christian and civilized world. Other sieges may have been more famous for their strategy and skill, but none more important than this in a moral, a religious, and a political point of view. It is a crisis in the course of events—a turning-point in the order of affairs. Barbarism must not gain or hold the ascendant. The intelligence, the science, and the prowess of England, as well as of Europe, must not succumb—no, not for an hour—to the black races of mankind, the acknowledged ignorance, brutality, and inferiority of the Asiatic hordes.

"A portion of our troops had escaladed the bastion walls close to the gate; others had scrambled up two large breaches in the walls and bastion near the Treasury. The remainder had entered at the gate itself, which had been blown open by one of our officers. The latter was really the hazardous duty of the day, and was most gallantly performed, though in broad daylight. The explosion party consisted of Lieutenants Home and Salkeld, of our corps, a few sappers, and a bugler of her Majesty's 52nd. On their nearing the gate they found it slightly open, with a number of muskets protruding at the opening. They were received with a volley, which killed and wounded a number. Still, however, they advanced, and fixed their powder bags on to the gate. Sergeant Carmichael, of the Sappers, tried to light the portfire attached to the bags, but was shot dead in the attempt. After him Sergeant Burness, of the Sappers, tried, but fell, pierced with three bullets. After Sergeant Burness, Salkeld tried, but fell into the ditch, shot in the leg, which is broken, and in the arm, which has been amputated. Sergeant Smith, of the Sappers, tried next, and he and Home between them happily succeeded without a wound. Seeing the portfire well a-light, the whole party jumped into the ditch, and the enemy, little dreaming of what had been done, continued firing till the explosion took place, so you may fancy what effect it took. Twenty of the mutineers strewed the road inside the gate, not one of whom would it have been possible to recognise, and this was the first sight that met my eyes at the gate. Little wonder that Donald refused to move; however, finally, he did, and got as far as the centre of the main guard, beyond which nothing would urge him. It was perfect agony keeping my seat, and I would have given worlds to dismount—but walking was out of the question; it was anything but an agreeable fix to be in. I felt how perfectly useless I was, and being the only mounted man there in the open space, fully accounted for the number of bullets whistling past my ears. There was no help for it, so I had to make my way back to camp as I best could, fully convinced of my own folly. You may imagine I had done neither leg nor ankle much good by the trip, and I had not many opportunities of seeing the remainder of the operations. We progressed slowly after this for two days; but having obtained possession of the magazine, containing about 130 guns, there could no longer be much doubt as to the result. The enemy, who had hitherto so stoutly resisted us, was losing heart, though he had succeeded in beating us back with loss and kept us at bay at two or three points. Daily and nightly large numbers continued to leave the city till the 20th, when we found ourselves in perfect possession, and the entire city evacuated. Immediately on receiving news of the evacuation, I started for the palace. You can have no conception of the scene of confusion—Riflemen, Goorkhas, Sikhs, and Punjabees quarrelling over their plunder, and such plunder to quarrel about! Old counterpanes, dirty blankets, tin and copper pots and pans, Sepoys' red coats, baskets, papers, packets of tinsel, bottles of rose water and otto of roses lying here, there, and everywhere, and the whole place in a state of squalor and wretchedness passing all description. On turning into the garden in front of Douglas's stables I found myself alone. I cannot tell you what a scene of desolation presented itself to my eyes. The garden had been an encamping ground of the Sepoys, and everything bore witness to its having been deserted in hot haste. The tents were still standing, but it seemed as if everybody had taken his armful out of the boxes, and, without caring for what might drop, had made the best of his way out of the place. The stillness, too, after the noise and riot that I had just quitted, was really appalling, and I stood at the foot of the stairs stupified as if with a heated atmosphere, and unable to move. I was roused by hearing three cheers from the apartments above me. . . . We have captured the King, his two sons and grandson—the three last (among whom was Mirza Mogul Bey) were most deservedly shot before they were brought into the city, where their bodies were exposed in the Chandney Chouk."*—*The Times*, Nov. 24, 1857.

* Another account:—"The gallantry with which the explosion party, under Lieutenants Home and Salkeld, performed the desperate duty of blowing up the Cashmere-gate, in broad daylight, will, I feel sure, be held to justify me in making special mention of it. The party was composed, in addition to the two officers named, of the following:—Sergeants John Smith and A. B. Carmichael, and Corporal Burgess, Sappers and Miners; Bugler Hawthorne, her Majesty's 52nd; 14 native Sappers and Miners, 10 Punjab ditto, musters covered by the fire of her Majesty's 60th Rifles. The party advanced at the double towards the Cashmere-gate. Lieutenant Home, with Sergeants Smith and Carmichael, and Havildar Mahor, all the Sappers leading and carrying the powder-bags, followed by Lieutenant Salkeld, Corporal Burgess, and a portion of the remainder of the party. The advanced party reached the gateway unhurt, and found that part of the drawbridge had been destroyed; but passing across the precarious footway supplied by the remaining beams, they proceeded to lodge their powder-bags against the gate. The

There can be no doubt as to the result. The personal feats of arms are the firstfruits of eventual success. One British soldier drives twenty Sepoys before him; five hundred or five thousand bayonets, under the victorious Havelock, utterly rout and disperse as many tens of thousands of the enemy. Taught by ourselves, the Sepoys are only good soldiers so long as they repeat the lessons they have been taught. They have no power of themselves to do more than this. They are deficient in the first principles of action, by which much is accomplished by a few manœuvres, and everything is worked out upon a well-digested plan, altered and adapted on the spur of the moment to the change of circumstances, instantly discerned by the ready eye of genius, and thoroughly understood by the practised hand of science. We are witnessing, upon a vast arena, the demonstration of the great theorem that knowledge is power, and that personal and national ascendancy depend, not upon the amount of brute force, but upon the wisdom, fortitude, and justice of the people by whom they are displayed.

---

